# Structural and Functional Basis for Inhibition of Erythrocyte Invasion by Antibodies that Target *Plasmodium falciparum* EBA-175

**DOI:** 10.1371/journal.ppat.1003390

**Published:** 2013-05-23

**Authors:** Edwin Chen, May M. Paing, Nichole Salinas, B. Kim Lee Sim, Niraj H. Tolia

**Affiliations:** 1 Department of Molecular Microbiology, Washington University School of Medicine, Saint Louis, Missouri, United States of America; 2 Protein Potential, Rockville, Maryland, United States of America; Seattle Biomedical Research Institute, United States of America

## Abstract

Disrupting erythrocyte invasion by *Plasmodium falciparum* is an attractive approach to combat malaria. *P. falciparum* EBA-175 (PfEBA-175) engages the host receptor Glycophorin A (GpA) during invasion and is a leading vaccine candidate. Antibodies that recognize PfEBA-175 can prevent parasite growth, although not all antibodies are inhibitory. Here, using x-ray crystallography, small-angle x-ray scattering and functional studies, we report the structural basis and mechanism for inhibition by two PfEBA-175 antibodies. Structures of each antibody in complex with the PfEBA-175 receptor binding domain reveal that the most potent inhibitory antibody, R217, engages critical GpA binding residues and the proposed dimer interface of PfEBA-175. A second weakly inhibitory antibody, R218, binds to an asparagine-rich surface loop. We show that the epitopes identified by structural studies are critical for antibody binding. Together, the structural and mapping studies reveal distinct mechanisms of action, with R217 directly preventing receptor binding while R218 allows for receptor binding. Using a direct receptor binding assay we show R217 directly blocks GpA engagement while R218 does not. Our studies elaborate on the complex interaction between PfEBA-175 and GpA and highlight new approaches to targeting the molecular mechanism of *P. falciparum* invasion of erythrocytes. The results suggest studies aiming to improve the efficacy of blood-stage vaccines, either by selecting single or combining multiple parasite antigens, should assess the antibody response to defined inhibitory epitopes as well as the response to the whole protein antigen. Finally, this work demonstrates the importance of identifying inhibitory-epitopes and avoiding decoy-epitopes in antibody-based therapies, vaccines and diagnostics.

## Author Summary

Malaria is a devastating parasitic disease that kills one million people annually. The parasites invade and multiply within red blood cells, leading to the clinical symptoms of malaria. Therefore, preventing red blood cell, entry through vaccines is an attractive approach to controlling the disease. Although widespread efforts to develop a vaccine by identifying and combining critical parasite blood-stage proteins are underway, a protective vaccine for malaria has proved challenging. This is in part because, while parasite proteins have the ability to elicit antibodies that prevent red blood cell invasion, these antibodies are a small proportion compared to the total collection of ineffective antibodies produced. We show an antibody that prevents red blood cell invasion targets regions of the critical parasite protein PfEBA-175 required for red blood cell engagement. We also show that an antibody that does not prevent red blood cell invasion recognizes a region far removed from important functional segments of PfEBA-175. Our work demonstrates that identifying the regions targeted by antibodies, and the mechanisms by which antibodies that prevent invasion function, should drive future vaccine development and studies measuring the effectiveness of current vaccine combinations.

## Introduction

PfEBA-175 is a *P. falciparum* parasite ligand that binds to its receptor GpA on erythrocytes in a sialic acid-dependent manner [1]–[5]. This binding event is necessary for erythrocyte invasion and consequently PfEBA-175 is a leading vaccine candidate [6]–[9]. PfEBA-175 has also paved the way for the concept and development of a *P. falciparum* receptor blockade vaccine [6], [7], [9]. Within PfEBA-175, region II (RII) is sufficient for GpA binding and is comprised of two Duffy Binding Like (DBL) domains [2], F1 and F2 [4].

Parasite entry into erythrocytes occurs in discrete steps: initial attachment, apical reorientation, tight junction formation, and invasion [10], [11]. During erythrocyte invasion, PfEBA-175 localized in micronemes is postulated to be exposed on the parasite, or cleaved resulting in a soluble fragment that allows binding to its receptor Glycophorin A [1], [3], [11], [12]. Structural studies suggest the RII regions of two PfEBA-175 molecules may dimerize around the glycosylated extracellular domains of GpA dimers on the erythrocyte during binding [13]. However, an *in vivo* demonstration of PfEBA-175 dimerization as it binds its receptor Glycophorin A, a dimer, during merozoite invasion of erythrocytes has yet to be reported. PfEBA-175 binds to GpA in a sialic acid-dependent manner as binding requires the sialic acid moieties of the O-glycans of GpA [4], [14]. Structural studies also identified sialic acid binding pockets in RII that are created by both monomers and are located close to the proposed dimer interface, suggesting that receptor binding and dimerization are intimately linked [13]. F1 and F2 each contain a β-finger that inserts into a cavity created by F2 and F1, respectively, of the opposite dimer. Upon binding, signaling occurs through PfEBA-175 to trigger rhoptry release and further maturation of the tight junction [15].

PfEBA-175 RII is recognized by antibodies in individuals with naturally acquired immunity [16]. In addition, antibody levels are associated with protection from malaria [16]–[18] although this association is not observed in groups with a low incidence of disease [19]. PfEBA-175 can be genetically deleted resulting in a switch to sialic acid-independent invasion [20], [21], and these alternate pathways may facilitate immune evasion [22]. However, a potent block in erythrocyte invasion is achieved when multiple pathways are targeted and targeting the PfEBA-175 sialic-acid dependent pathway is required for potent block in invasion in all combinations tested [7], [23]–[25]. Consistent with PfEBA-175 being the major chymotrypsin-resistant invasion pathway, anti-PfEBA-175 antibodies block invasion through sialic acid-dependent and -independent pathways [6], [21]. Lastly, antibodies recognizing regions of PfEBA-175 outside of RII may also play a role in immunity [18], [25], [26].

As RII is a natural target for immunity, antibody inhibition of PfEBA-175 mediated invasion has been the focus of intense study. R217 and R218 are IgG_1_ mouse monoclonal antibodies that recognize F2 and F1, respectively, and inhibit parasite growth to different extents [9]. R217 is the most potent *P. falciparum* inhibitory antibody developed to date with a growth inhibition IC_50_ of 10–100 µg/ml, while R218 is less potent with an IC_50_ greater than 1 mg/ml. Because of these properties, R217 has high therapeutic value, and both antibodies are important tools in defining the PfEBA-175/GpA interaction. Each antibody has a distinct mechanism, as the combination of R217 and R218 together block PfEBA-175 binding to erythrocytes to a greater extent than either antibody alone. While antibody inhibition of the critical PfEBA-175 invasion pathway has been appreciated, not all antibodies that recognize PfEBA-175 are inhibitory [9]. The molecular basis and mechanism for antibody inhibition is still unclear.

Here, we present two crystal structures of antibody Fab fragments from both R217 and R218 in complex with PfEBA-175, and identify the epitopes targeted by each antibody. Consistent with the location of each epitope, we show that R217, but not R218, directly prevents PfEBA-175 from engaging GpA in functional assays. We also confirm the strongly inhibitory R217 complex structure by small angle x-ray scattering (SAXS). Together, the results show that the potent R217 targets residues in PfEBA-175 that are functionally required for receptor-binding while the weakly-neutralizing R218 engages non-functional regions. We further demonstrate that R217 functions by directly blocking receptor binding while R218 may function through alternate mechanisms such as bivalent binding and steric hindrance. The two mechanisms of antibody inhibition are distinct and explain the additive effect when both antibodies are combined. These results elucidate the structural basis and mechanism for antibody inhibition and pave the way for future therapeutic design targeting PfEBA-175.

## Results

### R217 engages a conformational epitope in F2 of PfEBA-175

**Figure 1:**
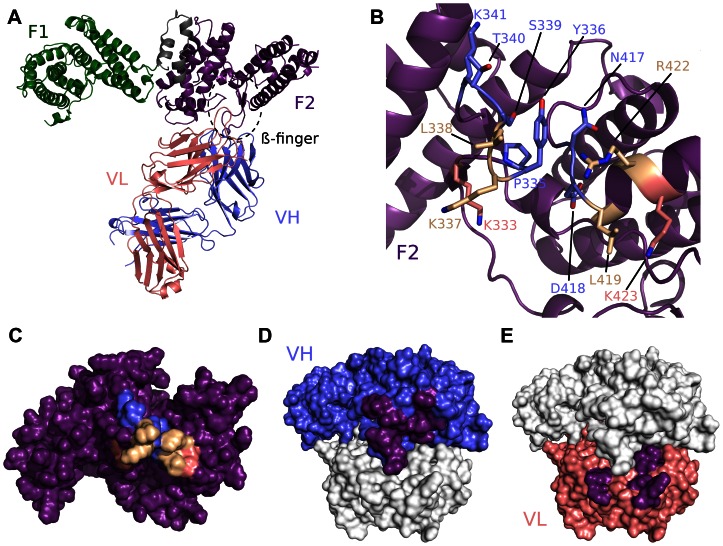
Crystal structure of RII/R217 Fab complex. (A) Overall structure of the RII/R217 Fab complex shown in ribbon representation. The F1 domain of RII is colored in green, the F2 domain of RII is colored purple. The Fab heavy chain (VH) is in blue and the light chain (VL) in pink. The location of F2 β-finger is circled in black. (B) Ribbon representation of F2 mapping the R217 epitope. Residues contacted by the Fab are show in stick. Residues contacted by the heavy chain are colored blue, residues contacted by the Fab light chain are colored pink, and residues contacted by both chains are in beige. Residues not contacted by the antibody are in purple. (C) Surface representation of F2 mapping the R217 epitope. Color scheme as in B. (D) Surface representation of the R217 Fab, mapping heavy chain residues (blue) that contact F2 (purple). The light chain is shown in white. (E) Surface representation of the R217 Fab, mapping light chain residues (pink) that contact F2 (purple). The heavy chain is shown in white.

We solved the crystal structure of the antibody Fab fragment from R217 in complex with RII of PfEBA-175 to a resolution of 4.5 Å (Table S1). At this resolution the residues in RII contacted by the antibody can be clearly identified as the backbone density is clear. However, inferences based on exact atom positions, particularly in side chains, should be made with caution. R217 binds to a conformational epitope primarily composed of two linear segments. The first linear segment is the β-finger within sub-domain 1 of F2 of RII including residues 331 to 341, and the second forms a loop and the end of a helix encompassing residues 417 to 423 (Figure 1B, Figure S1, Table S2). The structural basis of R217's conformational dependence and sensitivity to reducing conditions are disulfide bonds, including two in the β-finger, that retain the overall fold of the DBL domain bringing the linear segments together to form the conformational epitope [9]. All six CDR regions of the antibody engage with F2, particularly at the tip of the β-finger between residues 333 to 341 (PYKLSTK) which forms the central region of the epitope (Figure 1, Figure S1, and Table S2). The total buried surface area is 1394 Å^2^ and is comprised of 897 Å^2^ and 497 Å^2^ from the heavy and light chain, respectively (Figures 1C–F). A shape complementarity of 0.67 for this antibody-antigen interaction places it within the average range of 0.64–0.68, where perfect complementarity is 1.00 [27]. These binding parameters are consistent with typical values for antibody-antigen interactions. Binding of R217 to RII alters the hinge angle between F1 and F2 without changing the structure of individual domains (RII rmsd 2.08 Å, F1 rmsd 0.86 Å, F2 rmsd 0.57 Å) (Figure S1D). Thus, the inhibitory effects are not due to disruption of the DBL fold, but may be due to its physical location on RII and disruption of native protein function.

### Small angle x-ray scattering of the R217 complex

**Figure 2:**
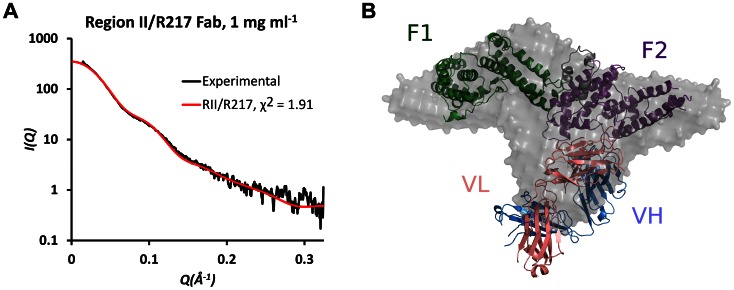
SAXS analysis of RII/R217 Fab. (A) Plot of scattering intensity (*I*) against scattering momentum (*Q*) and statistical fit of theoretical scatter from the RII/R217 Fab crystal structure (red line) with experimental SAXS profile (black). (B) Overlay of *ab initio* averaged reconstruction model of SAXS data with crystal structure. RII F1 domain is green, RII F2 domain is purple, the Fab heavy chain (VH) is blue and the Fab light chain (VL) is pink. The *ab initio* envelope is colored grey.

To support the structure presented above, SAXS analysis was performed on the RII/R217 complex (Figures 2A and 2B). SAXS returns a molecular envelope of species in solution, circumventing crystal packing artifacts, and is an independent method to determine the structure of the complex. The predicted scatter from the RII/R217 complex crystal structure fit the SAXS profile with a χ^2^ of 1.91. χ^2^ values less than 3 are indicative of a correct fit [28]. An *ab initio* averaged reconstruction model of the SAXS data further demonstrated the crystal structure matched the structure in solution. SAXS can also determine the molecular weight of samples. SAXS MOW [29] returned an estimated molecular weight for the RII/217 sample of 124.3 kDa (Figure S2). This value is consistent with the theoretical mass (123 kDa) of a complex of a monomer of RII (∼73 kDa) and a Fab monomer (∼50 kDa). Thus, the SAXS analysis strongly validates the R217 epitope.

### R218 targets an asparagine-rich surface loop

**Figure 3:**
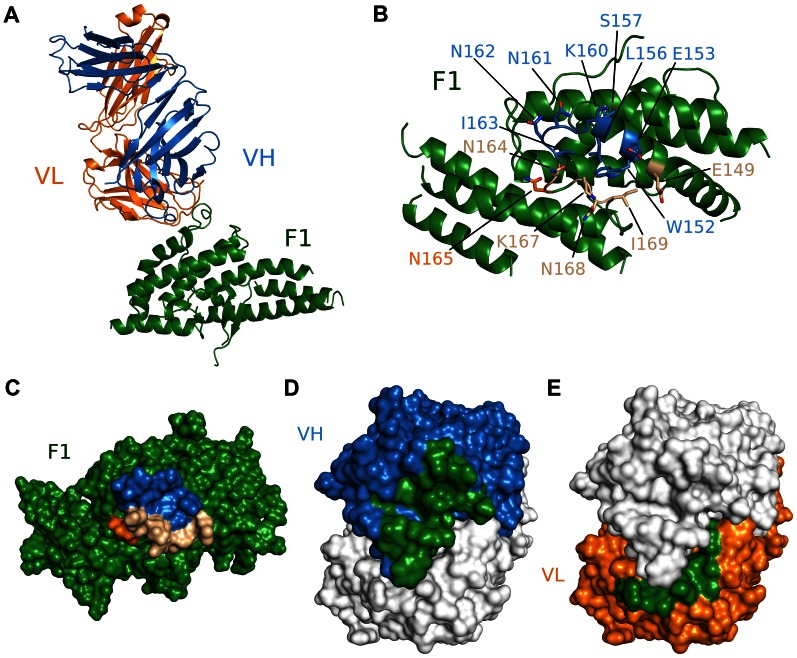
Crystal structure of F1/R218 Fab complex. (A) Overall structure of the F1/R218 Fab complex shown in ribbon representation. The F2 domain of RII is colored in green. The Fab heavy chain (VH) is in blue and the light chain (VL) in pink. (B) Ribbon representation of F1 mapping the R218 epitope. Residues contacted by the Fab are show in stick. Residues contacted by the heavy chain are colored blue, residues contacted by the Fab light chain are colored orange, and residues contacted by both chains are in beige. Residues not contacted by the antibody are in green. (C) Surface representation of F2 mapping the R217 epitope. Color scheme as in B. (D) Surface representation of the R218 Fab, mapping heavy chain residues (blue) that contact F2 (green). The light chain is shown in white. (E) Surface representation of the R218 Fab, mapping light chain residues (orange) that contact F2 (green). The heavy chain is shown in white.

We also obtained the crystal structure of the antibody Fab fragment from R218 in complex with the F1 domain of PfEBA-175 to a resolution of 2.4 Å (Table S1). R218 binds to a helix and loop between sub-domains 2 and 3 in F1 encompassing residues 149 to 169 (Figure 3A, Table S3). Residues 160 and 165 (KNNINN) form the central region of the epitope (Figures 3, Figure S3, and Table S3). The F1/R218 complex also has typical antibody-antigen binding parameters. The heavy and light chains contribute buried surface areas of 935 Å^2^ and 455 Å^2^, respectively, for a total of 1390 Å^2^ (Figure 3C–F). The interface has a greater-than-average shape complementarity at 0.72. No major structural perturbations in F1 occur upon antibody binding (rmsd 0.588 Å to unbound F1) (Figure S3D). Thus, the weakly inhibitory effect of R218 is not due to drastic structural changes in the DBL domains.

### Epitopes identified are critical for antibody binding

**Figure 4:**
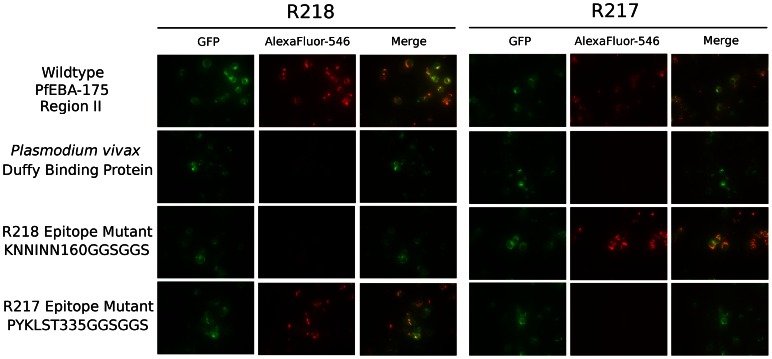
Antibody epitope identification by immunofluorescence assay. DBL domain proteins were surface expressed on HEK293 cells and probed with R217 or R218 as primary and Alexafluor-546 labeled anti-IgG_1_ as secondary. Green channel shows GFP tagged expressed protein. Red channel shows Alexafluor-546 labeled proteins on HEK293 cell surface. Merged channel shows overlap between green and red channels.

The structures reveal each antibody recognizes distinct epitopes. We used immunofluorescence binding assays to demonstrate that the epitopes identified are indeed the binding determinants for each antibody (Figure 4). Wildtype RII, epitope mutants or *P. vivax* Duffy Binding Protein (PvDBP) as the DBL negative control were expressed on the surface of mammalian cells [4], [30]. Recognition of surface expressed ligands by antibodies was assessed by fluorescence microscopy. For each epitope mutant, binding of the other antibody serves as a positive control for proper protein expression and folding. R217 bound wildtype RII and the R218 epitope mutant, but not PvDBP or the R217 epitope mutant. Likewise, R218 binds to wildtype RII and the R217 epitope mutant but not to PvDBP or the R218 epitope mutant. These studies demonstrate that in both cases, the correct epitope was identified by the structural analyses.

### R217 engages functional residues in RII while R218 does not

**Figure 5:**
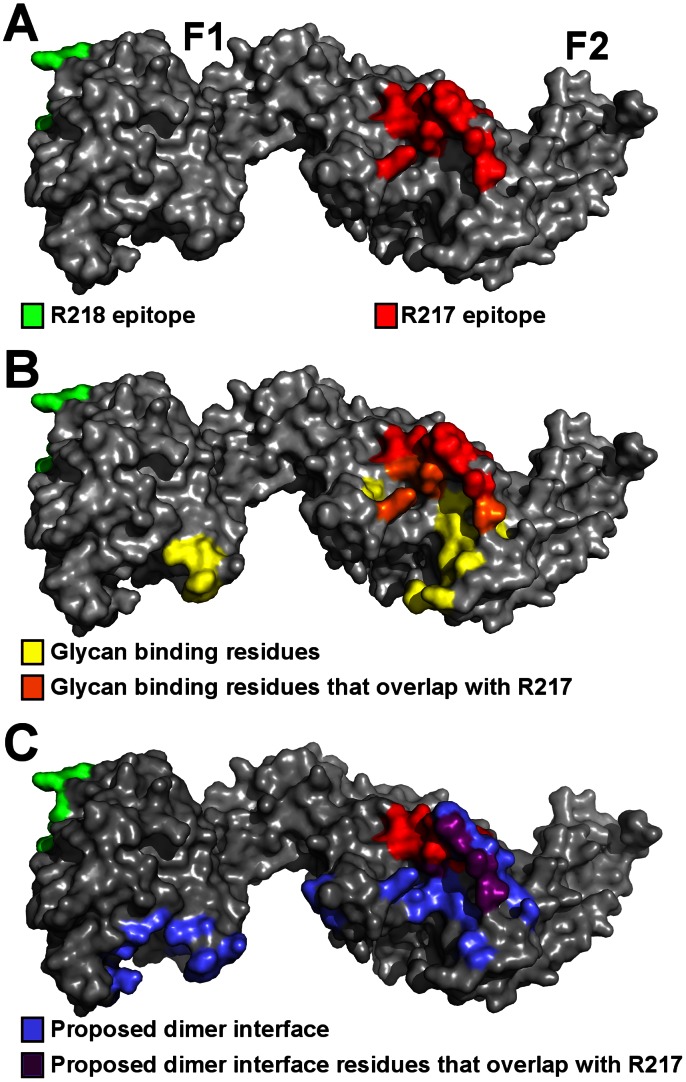
The R217 epitope overlaps with glycan binding residues and the proposed dimer interface, while the R218 epitope is far removed from these regions. (A) Surface representation of RII (grey) with the R217 (red) and R218 (green) epitopes. (B) The R217 epitope overlaps with glycan binding residues, including K341, N417 and R422 with demonstrated roles in erythrocyte binding [13]. The R218 epitope is located on the opposite face of RII away from glycan binding residues. Glycan binding residues are in yellow, glycan binding residues that overlap with the R217 epitope are in orange, the R217 epitope is in red, the R218 epitope is in green and RII is in grey. (C) The R217 epitope overlaps with proposed dimer interface residues while the R218 epitope is far removed from the proposed dimer interface. Proposed dimer interface residues are in blue, proposed dimer interface residues that overlap with the R217 epitope are in purple, the R217 epitope is in red, the R218 epitope is in green and RII is in grey.

R217 binds to a conformational epitope that includes the F2 β-finger (333–341), and a loop and helix (417–423). These regions include residues that are proposed to directly interact with glycans from GpA and have a demonstrated role in erythrocyte binding [13]. K341, N417 and R422 are proposed glycan binding residues that when mutated, reduce binding to erythrocytes [13]. These residues form part of the interface between R217 and RII (Figure 5A–B, Table S2) demonstrating R217 engages residues in RII that have a functional role in erythrocyte binding. Antibody binding therefore prevents direct receptor binding by blocking the heavily glycosylated GpA from accessing these binding pockets during tight junction formation. A secondary effect of R217 may be through modulation of RII dimerization as dimerization has been proposed to play a role in GpA engagement. The R217 epitope also encompasses residues (S338, T340, K341, N417) found at the proposed dimer interface (Figure 5C). Antibody binding may also prevent two RII molecules from coming in close proximity, preventing the F2 β-finger from inserting into the corresponding F1 cavity of the opposite anti-parallel monomer. This would lead to ablation of putative RII dimerization around a GpA pair that may be necessary for optimal EBA-175 binding to its receptor Glycophorin A.

On the other hand, R218 binds to an area far removed from functional receptor binding pockets and/or dimerization interface of RII (Figure 5A–C). Since residues in the R218 epitope have not been tested for function, we performed erythrocyte binding assays on residues in the R218 epitope. Mutation of six central residues in the epitope to a glycine-serine linker had no effect on erythrocyte binding (Figure S4). However, it is plausible that mutations to glycine or serine are insufficient to disrupt interaction with GpA. We therefore also mutated residues in the epitope to introduce bulky residues or charge reversal. Again, none of these changes had any effect on erythrocyte binding. Thus, R218 does not recognize residues that are functionally required for erythrocyte binding. This suggests R218 exerts its activity through mechanisms other than a direct block in receptor binding.

### R217 prevents RII from directly engaging GpA

**Figure 6:**
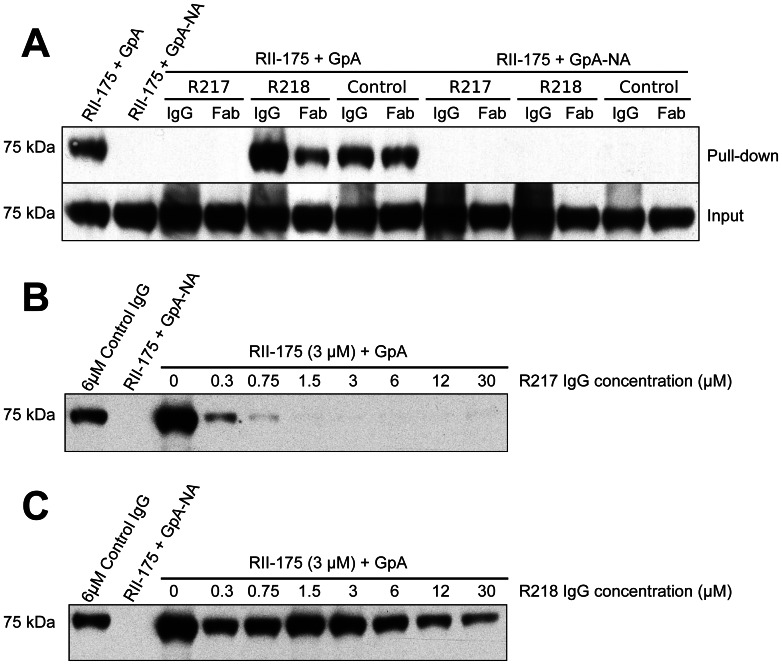
Direct antibody-inhibition of GpA binding by RII. (A) RII binds to GpA (lane 1), but not to neuraminidase treated GpA (GpA-NA – lane 2). Addition of R217 IgG or Fab prevents RII from binding GpA (lane 3 and 4). Neither R218 IgG (lane 5), R218 Fab (lane 6), control IgG (lane 7) nor control Fab (lane 8), block RII/GpA receptor binding. Binding is specific as neuraminidase treatment prevents binding in all cases (lanes 2 and 9–14). Note that an increased signal is observed with R218 IgG over R218 Fab (compare lanes 5 and 6) suggesting bivalent binding. This assay was performed with RII at 3 µM and antibody or Fab fragments at 6 µM. (B) Titration of R217 demonstrates the specificity of interaction as all concentrations around and above the available RII concentration (3 µM) prevents binding (lanes 6–10). At concentrations below the available RII binding occurs as not all the RII is bound by R217 (lanes 3–5). (C) Titration of R218 demonstrates that R218 is unable to directly prevent GpA binding at any concentration.

To test these mechanisms of inhibition, we performed inhibition studies of direct receptor binding in a protein-protein interaction assay (Figure 6A). These assays circumvent the caveats of GpA heterogeneity and the effects of GpA membrane embedding found when using whole erythrocytes in binding assays. Therefore, these studies allow for direct examination of antibody inhibition on receptor binding. Fully glycosylated His-tagged GpA captured on nickel-NTA beads was functional in binding recombinant RII. This interaction is specific as neuraminidase treatment of GpA, which removes the critical binding determinant sialic acid from GpA, completely ablates RII binding. Consistent with the mechanism that R217 interferes with residues necessary for receptor glycan binding, addition of the R217 antibody completely prevented RII binding to GpA. Furthermore, the Fab fragment of R217 alone was sufficient for inhibition demonstrating that bivalent antibody binding is not required for inhibition. To further demonstrate the specificity of inhibition we performed inhibition assays by titrating R217 (Figure 6B). R217 prevented GpA binding at all concentrations around and above the available RII concentration (3 µM). RII binding was observed at antibody concentration significantly lower than the available RII demonstrating the inhibitory effect is specific and is dependent on R217 concentrations comparable to the available RII.

### R218 allows for receptor engagement and may bivalently bind RII

In contrast, addition of R218 IgG or Fab fragments did not decrease the RII signal demonstrating R218 is unable to directly disrupt GpA binding (Figure 6A). Interestingly, the R218 IgG showed an increase in RII signal which was not observed for the Fab fragment. This result suggests that R218 antibody may bivalently cross-link several RII species while still allowing RII to contact GpA. Thus, R218 can form a ternary complex with GpA and RII, and even in the presence of R218, functional invasion can still occur. To ensure the lack of inhibition was not concentration dependent, we varied the concentration of R218 in the inhibition assay (Figure 6C). In contrast to R217, binding of RII to GpA was observed at all concentrations of R218 including those significantly greater than the available RII. This demonstrates that R218 does not result in a direct block in GpA binding.

## Discussion

The first ever structures of EBL ligands in complex with inhibitory antibodies presented here elucidates the dynamic process of receptor-ligand interactions during invasion. In addition, these findings provide an explanation of the difference in potency of R217 and R218. We demonstrate that R217 engages critical receptor-binding residues in PfEBA-175 with a demonstrated role in erythrocyte binding [13]. The functional regions include additional glycan binding residues and the proposed dimer interface of RII. In addition, R217 directly prevents GpA engagement by RII. Thus, we conclude the R217 directly blocks GpA binding as it engages functional residues in RII. On the other hand, the R218 epitope is far removed from proposed functional regions and mutation of residues in the epitope have no effect on erythrocyte binding. R218 is also unable to directly prevent GpA binding at any concentration tested. Thus, we conclude R218 does not directly inhibit GpA binding by RII as it engages non-functional regions. While this study was under review, a phage display approach to map epitopes targeted by anti-PfEBA175 monoclonal antibodies identified residues contacted by R217 [31]. The structural and mapping studies presented here are consistent with the phage display results.

These results allow for the proposal of putative models of action (Figure S5). PfEBA-175 binds the sialic acid glycans of GpA and binding has been proposed to dimerize around GpA [13]. R217 inhibits erythrocyte invasion by engaging critical receptor-binding residues and the proposed dimerization interface in PfEBA-175. R217 binding therefore prevents access to receptor-binding residues necessary for GpA recognition. A secondary effect of R217 may be to prevent dimerization as the R217 epitope is coincident with the proposed dimer interface. However, further studies are necessary to demonstrate antibody effects on oligomeric state upon receptor binding.

In contrast, R218 does not prevent receptor binding and can form a ternary complex with RII and GpA. Our results suggest R218 may bivalently bind RII, and bivalent antibody binding may lead to agglutination of ligands. The IC50 for R218 is greater than 1 mg/ml and is therefore weakly inhibitory [9]. We propose that at antibody concentrations below the IC50, R218 engages PfEBA-175 but this binary complex can still bind GpA allowing productive receptor-ligand interaction and parasite invasion. At antibody concentrations greater than the IC50, the bivalent R218 antibody may cross-link multiple RII molecules before a productive interaction can take place, preventing invasion, and explaining the weakly inhibitory effect in growth inhibition. Bivalent binding by R218 in close proximity to the erythrocyte membrane could also sterically prevent GpA binding in agreement with previous findings that R218 blocks RII binding to erythrocyte surfaces at high antibody concentrations [9].

The two different mechanisms may also explain how R217 and R218 act cooperatively to block parasite growth [9]. R217 directly blocks receptor binding and may prevent dimerization while R218 bivalently binds RII. The bivalent binding may increases exposure of the R217 epitope to enhance R217 binding resulting in a cooperative inhibition.

These structural studies show that the epitopes identified in this study are ideal candidates for therapeutic design due to their flexibility and conformability as surface exposed loops and immunogenic nature when immunized. However, the implications of this study go beyond simply identifying therapeutic epitopes within PfEBA-175. All current malaria vaccines and antibody-based therapies are based on previously identified receptor binding domains, immunogenic epitopes or host immunogenic response to foreign antigen [32], [33]. The leading candidate, RTS,S, is composed of dual B- and T-cell immunodominant epitopes [32], [34]. The studies presented here provide a proof-of-concept that as with other infectious diseases, targeting critical mechanisms of protein function may provide potent protection [35]–[37]. Both antibodies were generated as a result of highly immunogenic epitopes, and both have high apparent affinity towards their targets. However, only R217, which binds to a functional region of PfEBA-175, possess potent activity against *P. falciparum*. R218, which binds to a surface loop with no known function, is essentially non-inhibitory. R218 has a hundred fold higher apparent affinity (0.0153 nM) for PfEBA-175 than R217 (1.84 nM) [9] suggesting affinity alone is not a good correlate for inhibitory potential. Comparing R217 and R218 impresses upon the necessity of targeting protein mechanisms when using antibody therapeutics. Lastly, identifying multiple methods of inhibition will aid in the development of combination antibodies therapies leading to synergistic blocks in invasion.

The approach presented here of defining the molecular and mechanistic basis of antibody inhibition can also be adapted to other EBL family members such as PvDBP and PfEBA-140. The erythrocyte receptors for these ligands are known, and the mechanism by which receptor-binding occurs are being defined [38]–[41]. Potent inhibitory antibodies could be generated in each case to block invasion.

Finally, R218 highlights an issue concerning parasite immune evasion [42]. Several *Plasmodium* antigens contain extensive asparagines rich repeats with high degeneracy and potential cross-reactivity. This network of cross-reacting epitopes has been proposed to dampen an effective immune response through generation of non-protective antibodies and decreased efficacy of affinity maturation [42], [43]. R218's KNNINN epitope is a highly antigenic asparagine rich region. Based on its structural and functional properties, the results presented suggest R218 fits the profile of an antibody targeting a decoy site. Further studies are necessary to examine the potential role of this site in immune evasion. The results presented here are representative of the challenges faced when vaccinating with recombinant *Plasmodium* proteins that contain immunoevasive epitopes and serve to underscore the importance of identifying critically important functional epitopes like those of R217.

In conclusion, we present the crystallographic mapping of monoclonal antibodies against functional epitopes on a protein that is critical for parasite survival. The structures provide a glimpse into the mechanistic process of erythrocyte invasion through the PfEBA-175/GpA pathway at the tight junction. The studies presented here establish a rationale for the creation of vaccines against malaria parasites through epitope targeting as has been described in other systems [44]–[47]. This work also demonstrates the importance of defining epitopes in the context of function to develop strategies that will synergistically block invasion. In addition, these studies have implications for diagnostics aimed at measuring the efficacy of vaccines and for future studies to assess antigen selection and combination of multiple antigens leading to a viable blood-stage vaccine [6], [48]. Immunization with whole protein domains results antibodies that recognize multiple epitopes, yet only inhibitory epitopes such the epitope for R217 lead to antibodies that can productively block in invasion. Therefore, careful assessment of the antibody response not just to the entire protein domain, but to inhibitory epitopes is warranted to accurately assess the efficacy of a single and combination antigen trials and studies. Finally, the results presented here introduce the idea of targeting the mechanism of protein function for intervention and its necessity in creating potent therapeutics against malaria.

## Materials and Methods

### Protein expression and purification

RII was obtained as previously described [13] or by oxidative refolding. F1-175 was obtained by oxidative refolding. Inclusion bodies expressed in *E. coli* were solubilized in 6 M guanidinium hydrochloride and refolded via rapid dilution in 400 mM L-arginine, 50 mM Tris pH 8.0, 10 mM EDTA, 0.1 mM PMSF, 2 mM reduced glutathione, and 0.2 mM oxidized glutathione. Refolded protein was captured on SP Sepharose Fast Flow resin (GE Healthcare). Eluted protein was additionally purified by sequential size exclusion chromatography (GF200) and ion exchange chromatography (HiTrapS). Protein was finally buffer exchanged into 10 mM HEPES pH 7.4, 100 mM NaCl with size exclusion chromatography. The signal peptide and extracellular domain of Glycophorin A were cloned into plasmid pHLFcHis [49]. Glycosylated Glycophorin A was obtained by transient transfection in HEK293F cells. Five days post-transfection, GpA was captured by Q-resin followed by Ni-NTA affinity chromatography and purified by size exclusion chromatography in 10 mM HEPES pH 7.4, 100 mM NaCl. Neuraminidase-treated GpA was obtained by incubating GpA with neuraminidase from *Clostridium perfringens* (Sigma) for 2 hours at 37°C.

### Antibody digestion and Fab purification

Fab fragments were generated from IgG using immobilized papain resin and purified with protein A beads as per manufacturer's instructions (Thermo Scientific). The eluted Fab fractions were purified by size exclusion chromatography in 10 mM HEPES pH 7.4, 100 mM NaCl.

### Protein crystallization and data collection

RII/R217 complexes were created by mixing RII and Fab in a 1∶2 molar ratio and incubated at 4° Celsius for 2 hours. Complex was purified by size exclusion chromatography with the buffer 10 mM Tris pH 8.0, 100 mM NaCl. RII/R217 crystals were grown by hanging-drop vapor diffusion by mixing 1 µl of protein solution at 20 mg/ml and 1 µl of reservoir containing 0.3 M magnesium chloride, 8% benzamidine hydrochloride, 7.5% glycerol and 18% PEG 3350. RII/R217 crystals grew as clusters from which a single crystal had to be separated. Crystals were flash frozen in liquid nitrogen and RII/R217 data were collected to a resolution of 4.5 Å at beamline X26C, Brookhaven National Laboratory and processed with XDS [50]. Extensive efforts were made to improve the diffraction limits of these crystals including varying crystal growth conditions (component concentrations, growth setup, temperature and time), testing a variety of crystal growth, additives and annealing. However, none of these approaches improved diffraction. Attempts to obtain a higher diffraction data set by protein engineering also did not meet with success. R217 Fab in complex with various N- and C- terminal truncations of RII, and also the F2 domain only were purified. However, no crystals were obtained with these samples. Thus, the available 4.5 Å data was used to solve the RII/R217 structure.

F1/R218 complexes were created by mixing ligand and Fab in a 1.05∶1 molar ratio and incubated at 4° Celsius for 30 minutes. Complex was purified by size exclusion chromatography with the buffer 10 mM HEPES pH 7.4, and 100 mM NaCl. F1/R218 crystals were grown by hanging-drop vapor diffusion by mixing 1 µl of protein solution at 12 mg/ml and 1 µl of reservoir containing 0.1 M phosphate-citrate pH 4.2, 0.4 M lithium sulfate and 30% PEG 1000. Large single crystals of F1/218 grew within a week and were flash frozen in liquid nitrogen. Data were collected to a resolution of 2.4 Å at beamline 19-ID at the Advanced Photon Source, Argonne National Laboratory and processed with XDS [50].

### Structure solution and analysis

The F1/R218 structure was solved by molecular replacement in PHASER [51] using the F1 domain from 1ZRL and Fab domain from 3NZ8 as starting models. Manual rebuilding in COOT [52] and refinement in PHENIX [53] led to a final refined model with final R-factor/R-free of 20.88%/25.07% with good geometry as reported by MOLPROBITY [54]. The MOLPROBITY score of 1.29 places this structure in the top 100^th^ percentile of structures 2.20–2.70 Å. 95.5% of residues lie in favored, 4.5% of residues lie in additionally allowed, and 0% lie in disallowed regions of the Ramachandran plot.

The RII/217 structure was solved by molecular replacement in PHASER [51] using 1ZRL and homology model for the Fab derived from MODELLER [55]. Two copies of the complex were found in the asymmetric unit and tight NCS constraints were used throughout the refinement. Initial rigid body refinement in PHENIX [53] resulted in R-factor/R-free of 34.12%/35.72%. Due to the resolution of the data, careful refinement using DEN refinement in CNS [56] was performed. Subsequent repeated rounds refinement in PHENIX [53] with tight geometric constraints and manual building in COOT [52] resulted in final R-factor/R-free of 23.10%/28.47%. These low R-factors coupled with excellent geometric statistics as reported by MOLPROBITY [54] indicated structure refinement was complete. The MOLPROBITY score of 1.74 places this structure in the top 100^th^ percentile of structures 3.25–4.75 Å. 93.65% of residues lie in favored, 6.35% of residues lie in additionally allowed, and 0% lie in disallowed regions of the Ramachandran plot. Interface residues were identified by PISA [57].

### Antibody sequencing

Hybridoma cell lines MRA-711 and MRA-712 were obtained from MR4/BEI resources. Cell lines were grown in DMEM with 20% fetal bovine serum, 50 U/ml penicillin, 50 µg/ml streptomycin, 2% L-glutamine, 1% sodium pyruvate, and 0.1% β-mercaptoethanol. RNA was extracted using QiaShredder (Qiagen) and RNeasy Mini Kit (Qiagen). 5′ RACE Kit (Invitrogen) was used to obtain cDNA for the Fab region heavy and light chains. RACE primers for the heavy chain were: GSP1 for IgG_1_ isotype is TGCATTTGAACTCCTTGCC and GSP2 for IgG_1_ isotype is CTTTGGGGGGAAGATGAAG. RACE primers for the light chain were: GSP1 for Cκ is CACTCATTCCTGTTGAAGC and GSP2 for Cκ is CTTGTGAGTGGCCTCACAGG. cDNA was TOPO cloned (Invitrogen) and sequenced.

### Small-angle X-ray scattering

Data for RII/217 at 1 mg ml^−1^ was collected at the SIBYLS beamline 12.3.1 at the ALS using standard procedures [58]. Radiation damage was assessed by overlaying short exposures using PRIMUS [59]. As no radiation damage was observed the short and long exposures were merged in PRIMUS. The experimental profile was compared to the RII/217 structure using CRYSOL [60]. *Ab initio* model generation was performed in DAMMIF [61] and the filtered average envelope of ten models was obtained by DAMAVER [62]. SUPCOMB20 [63] was used to align structures and SAXS reconstructions. The molecular weight estimate was obtained using SAXS-MOW [29] on the merged dataset.

### Immunofluorescence assay

The R217 epitope mutant was created by mutating the sequence at the center of the epitope beginning at P335 and ending T340 (PYKLST) to a glycine-serine linker (GGSGGS). The R218 epitope mutant was created by mutating the sequence at the center of the epitope beginning K160 and ending N165 (KNNINN) to a glycine-serine linker (GGSGGS). Adherent HEK293T cells were grown in 6-well tissue culture plates and transfected with DNA containing gene sequences of either wildtype PfEBA-175 RII, PvDBP RII, R217 epitope mutant, or R218 epitope mutant in the pRE4 vector [4]. The cells were probed with 10 µg/ml of R217 or R218, washed, probed with 1 µg/ml Alexafluor-546 labeled α-mouse IgG_1_ (Invitrogen), washed, and imaged with a fluorescent microscope.

### RII/Glycophorin A binding assay

Fifty microliters of Ni-NTA beads slurry was used to incubate 8 µM of either untreated or neuraminidase treated 6×His-tagged GpA together with 3 µM purified RII in the presence or absence of various concentrations of R217, R218, R217 Fab or R218 Fab in binding buffer (50 mM Tris-HCl pH 8.0, 200 mM NaCl, 0.1% Triton X-100, and 20 mM imidazole), at the final volume of 500 µl at 4 C. The complexes were eluted off the Ni^2+^ beads with 50 µl of elution buffer (50 mM Tris-HCl pH 8.0, 100 mM NaCl, 500 mM Imidazole) and equal volume of protein sample buffer was added. The eluted complexes were resolved by SDS-PAGE, transferred, and immunoblotted with anti-RII (R218 antibody), followed by secondary antibody conjugated to horseradish peroxidase. Immunoblots were developed with ECL-PLUS (GE Healthcare) and detected by film.

### Functional studies

Rosette assays were performed as previously described [4], [13], [38], [40], [41].RII expressed as a C-Terminal GFP fusion on HEK-293T cells. Mutants were created using the QuikChange method and verified by plasmid sequencing. HEK-293T cells were transfected with plasmid DNA in polyethyleneimine. 16 hours post transfection erythrocyte binding of individual constructs was assayed by incubating transfected cells with normal human erythrocytes at 2% hematocrit for 30 min. Unbound erythrocytes were removed by washing 3 times with Phosphate Buffered Saline (PBS). The number of rosette positive cells over the number of GFP positive cells, normalized to wild type binding, yielded the binding percentage. Binding phenotypes were quantified over at least nine fields of view for each construct. Images were obtained using a Zeiss LSM 510 META Laser Scanning Microscope with an LD Achroplan 20× Korr DIC objective and counting was performed in ImageJ.

### Accession numbers

The atomic coordinates and structure factors for the structures have been deposited in the protein data bank with accession numbers 4K4M and 4K2U.

## Supporting Information

Figure S1 Interface residues between RII and R217Fab and RMSD analysis of RII/R217Fab complex DBL domains. (A) Contacting residues between RII (purple) and the light chain of R217Fab (pink). The R217Fab heavy chain is in white. (B) Contacting residues between RII (purple) and the heavy chain of R217Fab (blue). The R217Fab light chain is in white (C) 2Fo-Fc electron density map contoured at 1 sigma (blue mesh) of interface between RII (purple) and R217Fab (light chain – pink, heavy chain - blue). (D) Top left panel: Alignment of F1 from RII/R217Fab complex (green) with unbound F1 (white). Bottom left panel: Alignment of F2 from RII/R217Fab complex (purple) with unbound F2 (white). Top right panel: Alignment of RII from RII/R217Fab complex (F1 – green, F2 – purple) with unbound RII (white). Bottom right panel: Alignment of F1 from RII/R217Fab complex (F1 – green, F2 – purple) with F1 from unbound RII (white).
(TIFF)

Figure S2 SAXS-MOW analysis of the RII/R217 SAXS data return an estimated molecular weight of 124.3 kDa.
(TIFF)

Figure S3 Interface residues between F1 and R218Fab and RMSD analysis of F1/R218Fab DBL domain. (A) Contacting residues between F1 (green) and the light chain of R218Fab (orange). The R217Fab heavy chain is in white. (B) Contacting residues between F1 (green) and the heavy chain of R218Fab (blue). The R217Fab light chain is in white. (C) 2Fo-Fc electron density map contoured at 1 sigma (blue mesh) of interface residues between F1 (green) and R218Fab (light chain – orange, heavy chain - blue). (D) Alignment of F1 from the F1/R218Fab complex (green) with unbound F1 (white).
(TIFF)

Figure S4 Residues in the R218 epitope are not required for erythrocyte binding. (A) and (B) surface expression of GFP tagged RII on HEK293 cells demonstrates that mutants with changes in the F1 epitope binds erythrocytes as well as wildtype. HEK293 cells are the larger adherent cells, erythrocytes are smaller and appear as rosettes when bound. Left panel is bright field, center panel shows GFP expressing cells, and right panel is merge of left and center panels. (A) wildtype and a mutant where six residues at the center of the F1 epitope are mutated to a glycine-serine linker. (B) Individual residues in the F1 epitope were mutated to bulky or charge reversal residues in an attempt to introduce drastic changes. (C) and (D) show percentage of cells expressing RII that bind erythrocytes relative to wildtype for the mutants shown in (A) and (B) respectively.
(TIFF)

Figure S5 These results allow for the proposal of putative models of action. (A) RII has been proposed to dimerize around two GpA molecules on the erythrocyte surface to allow for parasite invasion. (B) R217 binds critical functional regions in RII and prevents direct engagement with GpA to block parasite invasion. A secondary effect for R217 may be to prevent dimerization. (C) At R218 concentrations below the IC50 a homo-dimeric ternary complex of R218 with RII and GpA is formed allowing for parasite invasion. (D) At R218 concentrations greater than the IC50, R218 bivalently binds RII at the parasite surface and prevents invasion. Glycophorin A is in red, PfEBA-175 F1 is in green/light green, PfEBA-175 F2 is in purple/light purple, the regions of PfEBA-175 outside RII are in dark blue, R217 light and heavy chain are in pink and slate blue respectively, R218 light and heavy chain are in orange and blue respectively, the IgG Fc is shown in white and lipid membranes are colored in light blue and yellow.
(TIFF)

Table S1 Data collection and refinement statistics
(PDF)

Table S2 RII-175/R217 interface residues defined by PISA [51]
(PDF)

Table S3 F1/R218 interface residues as defined by PISA [51]
(PDF)
