# Supplementary figures and images for: Structural and Functional Basis for Inhibition of Erythrocyte Invasion by Antibodies that Target Plasmodium falciparum EBA-175
Source: PLoS Pathog. 2013 May 23;9(5):e1003390. doi: 10.1371/journal.ppat.1003390 (PMC3662668; doi:10.1371/journal.ppat.1003390)

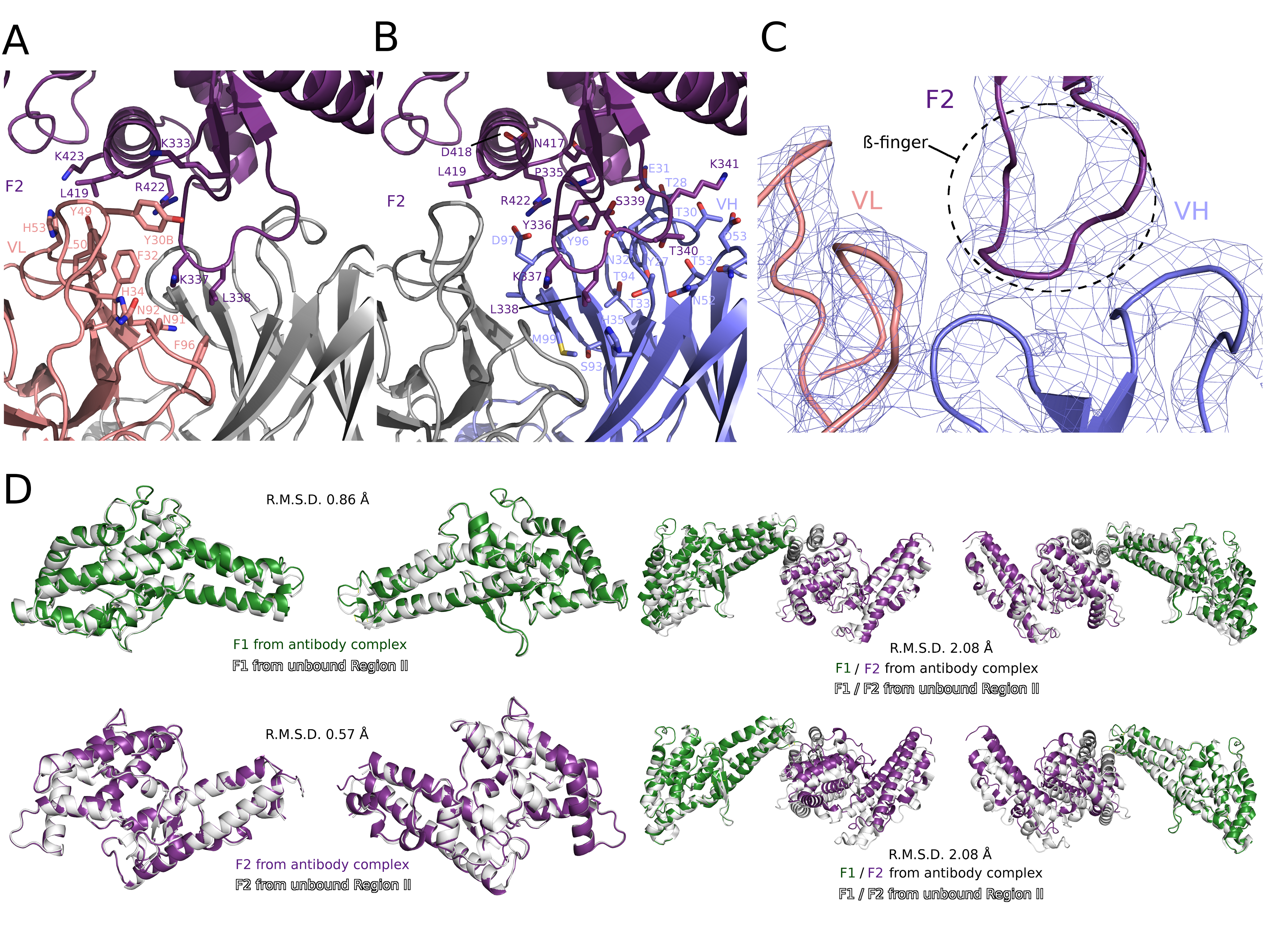

Supplement: Figure S1 — Interface residues between RII and R217Fab and RMSD analysis of RII/R217Fab complex DBL domains. (A) Contacting residues between RII (purple) and the light chain of R217Fab (pink). The R217Fab heavy chain is in white. (B) Contacting residues between RII (purple) and the heavy chain of R217Fab (blue). The R217Fab light chain is in white (C) 2Fo-Fc electron density map contoured at 1 sigma (blue mesh) of interface between RII (purple) and R217Fab (light chain – pink, heavy chain - blue). (D) Top left panel: Alignment of F1 from RII/R217Fab complex (green) with unbound F1 (white). Bottom left panel: Alignment of F2 from RII/R217Fab complex (purple) with unbound F2 (white). Top right panel: Alignment of RII from RII/R217Fab complex (F1 – green, F2 – purple) with unbound RII (white). Bottom right panel: Alignment of F1 from RII/R217Fab complex (F1 – green, F2 – purple) with F1 from unbound RII (white). (TIFF) [file ppat.1003390.s001.tif]

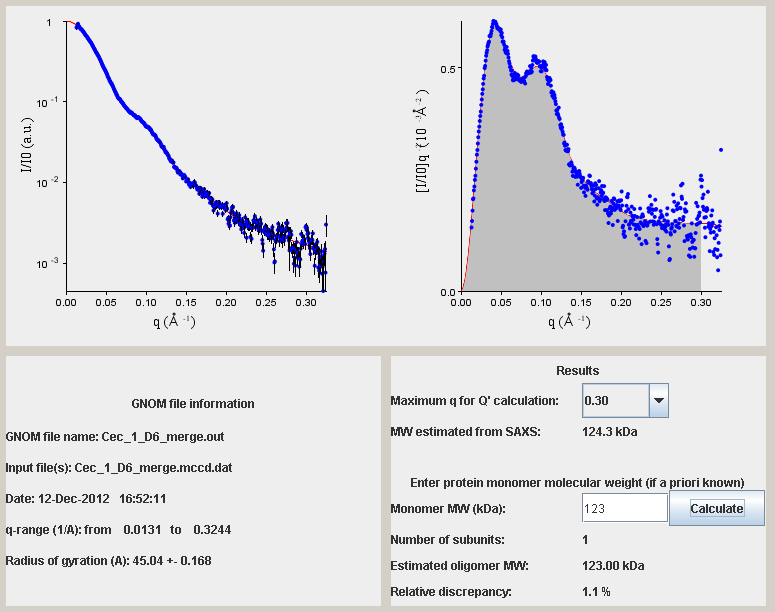

Supplement: Figure S2 — SAXS-MOW analysis of the RII/R217 SAXS data return an estimated molecular weight of 124.3 kDa. (TIFF) [file ppat.1003390.s002.tif]

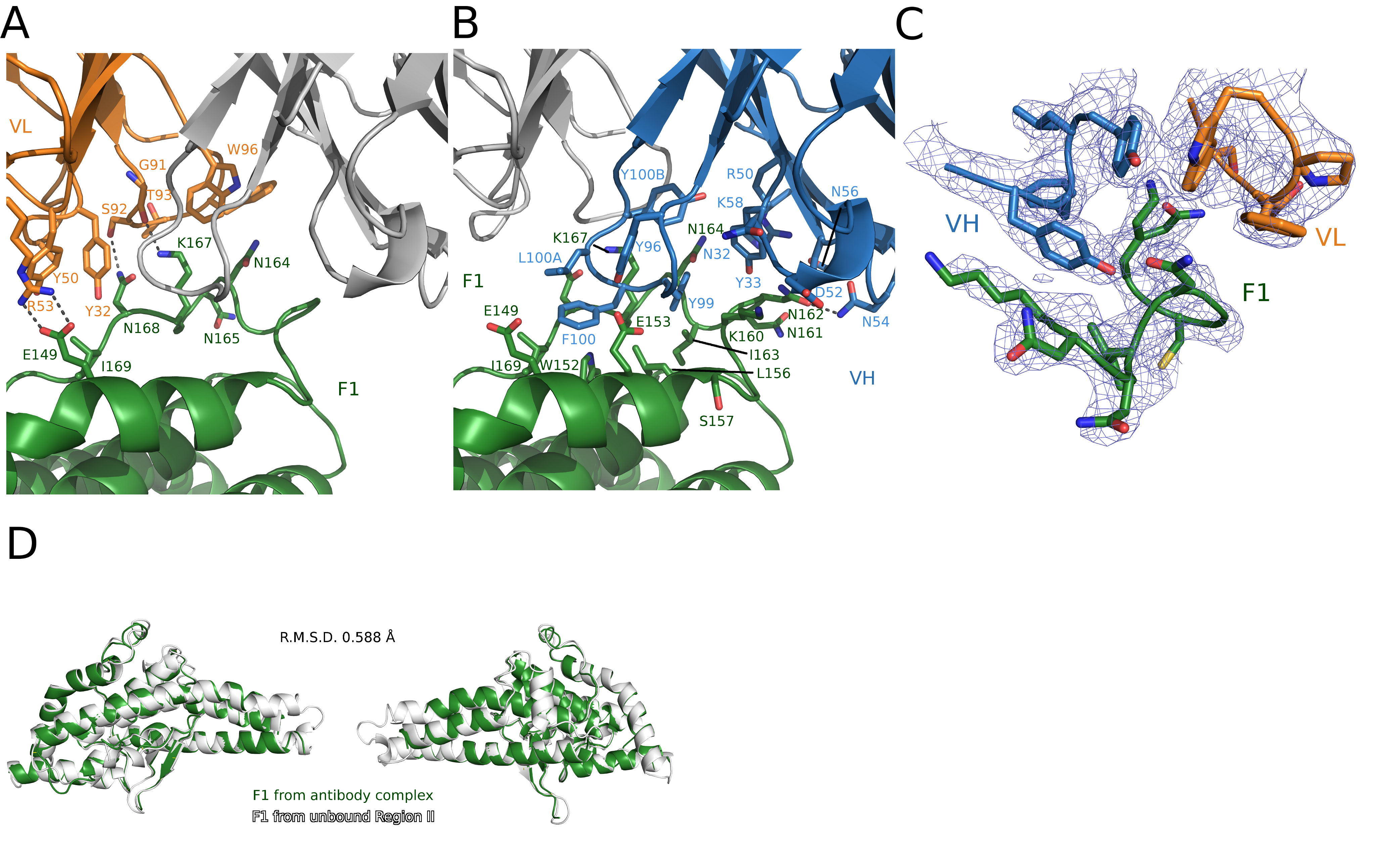

Supplement: Figure S3 — Interface residues between F1 and R218Fab and RMSD analysis of F1/R218Fab DBL domain. (A) Contacting residues between F1 (green) and the light chain of R218Fab (orange). The R217Fab heavy chain is in white. (B) Contacting residues between F1 (green) and the heavy chain of R218Fab (blue). The R217Fab light chain is in white. (C) 2Fo-Fc electron density map contoured at 1 sigma (blue mesh) of interface residues between F1 (green) and R218Fab (light chain – orange, heavy chain - blue). (D) Alignment of F1 from the F1/R218Fab complex (green) with unbound F1 (white). (TIFF) [file ppat.1003390.s003.tif]

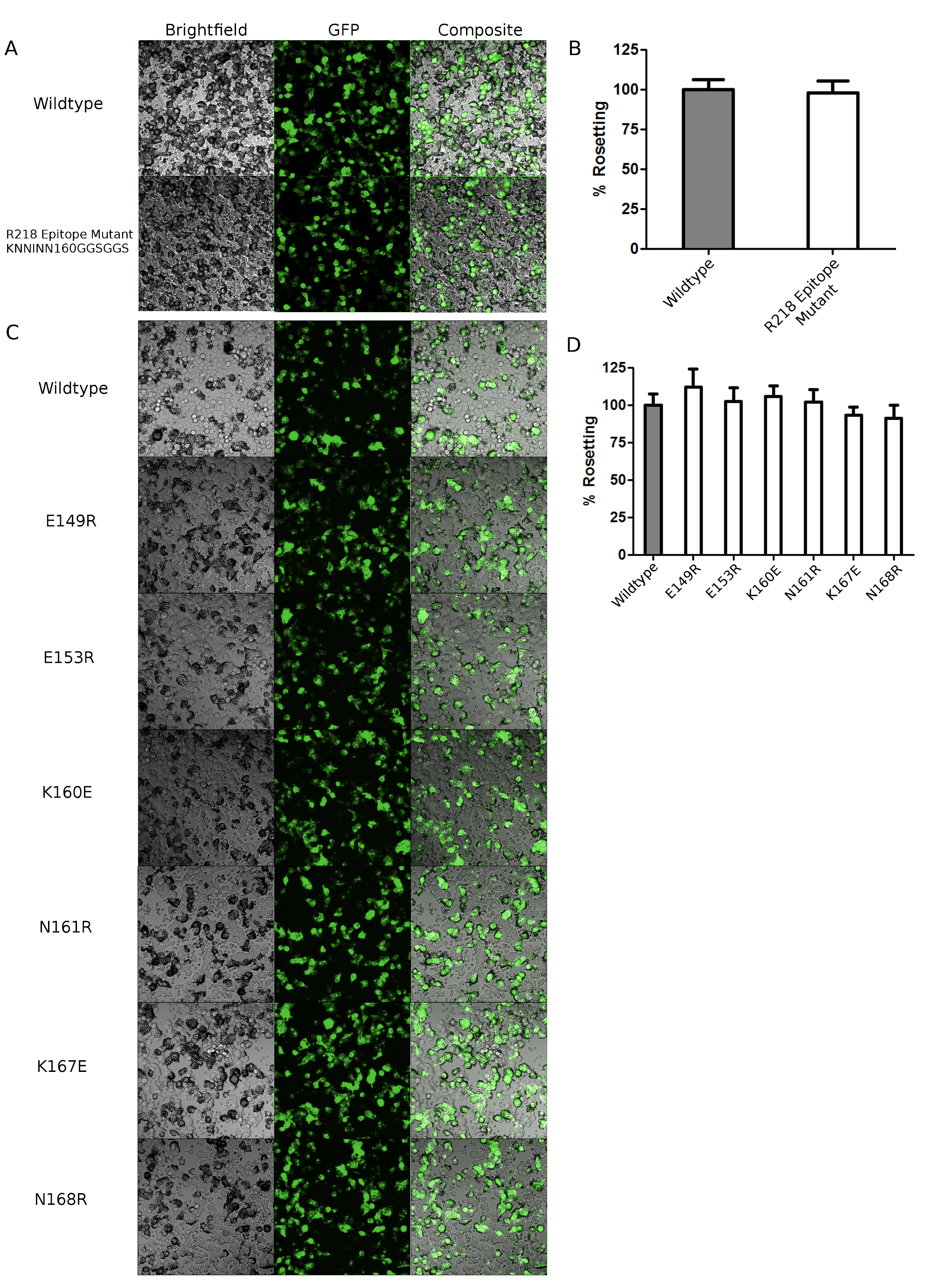

Supplement: Figure S4 — Residues in the R218 epitope are not required for erythrocyte binding. (A) and (B) surface expression of GFP tagged RII on HEK293 cells demonstrates that mutants with changes in the F1 epitope binds erythrocytes as well as wildtype. HEK293 cells are the larger adherent cells, erythrocytes are smaller and appear as rosettes when bound. Left panel is bright field, center panel shows GFP expressing cells, and right panel is merge of left and center panels. (A) wildtype and a mutant where six residues at the center of the F1 epitope are mutated to a glycine-serine linker. (B) Individual residues in the F1 epitope were mutated to bulky or charge reversal residues in an attempt to introduce drastic changes. (C) and (D) show percentage of cells expressing RII that bind erythrocytes relative to wildtype for the mutants shown in (A) and (B) respectively. (TIFF) [file ppat.1003390.s004.tif]

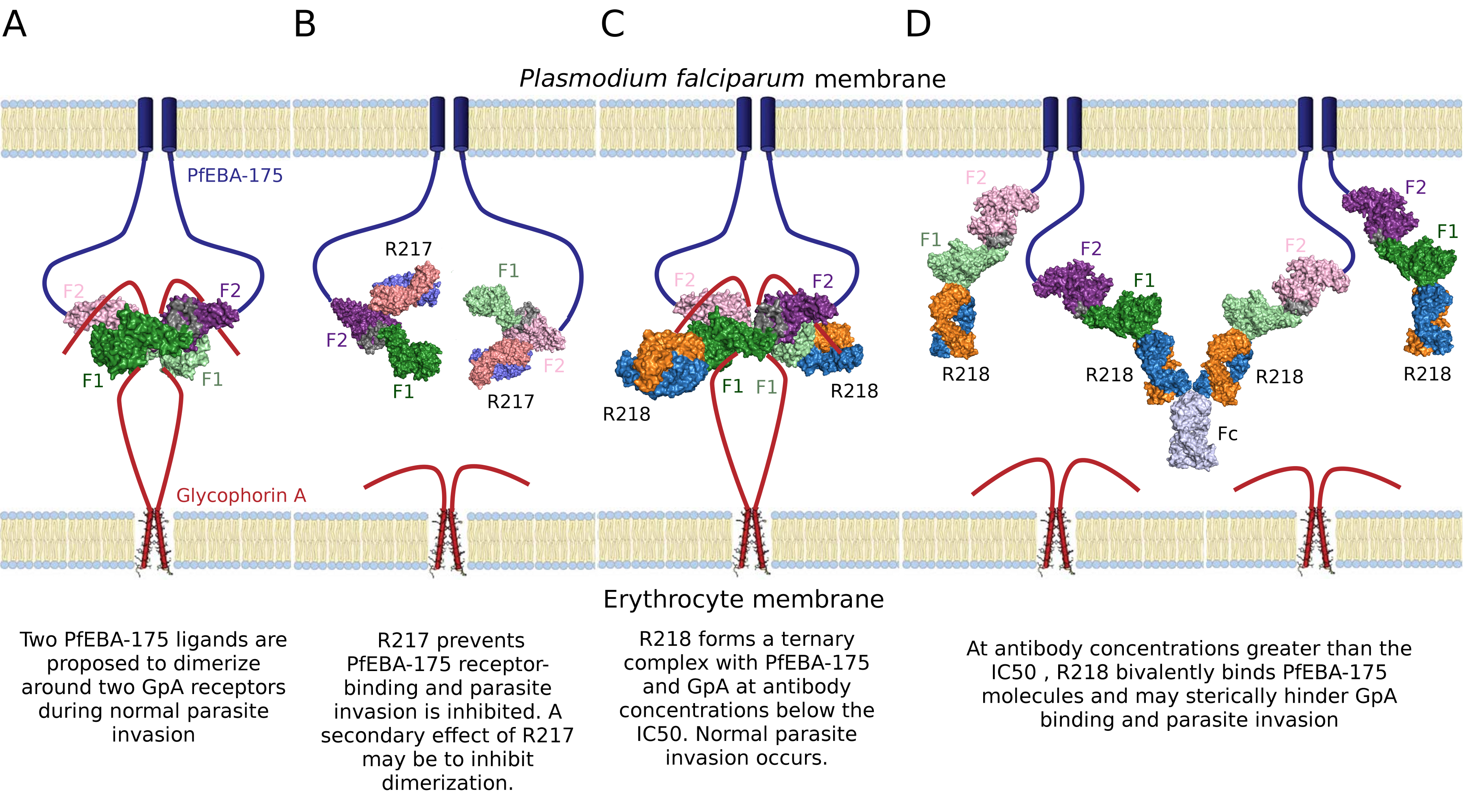

Supplement: Figure S5 — These results allow for the proposal of putative models of action. (A) RII has been proposed to dimerize around two GpA molecules on the erythrocyte surface to allow for parasite invasion. (B) R217 binds critical functional regions in RII and prevents direct engagement with GpA to block parasite invasion. A secondary effect for R217 may be to prevent dimerization. (C) At R218 concentrations below the IC50 a homo-dimeric ternary complex of R218 with RII and GpA is formed allowing for parasite invasion. (D) At R218 concentrations greater than the IC50, R218 bivalently binds RII at the parasite surface and prevents invasion. Glycophorin A is in red, PfEBA-175 F1 is in green/light green, PfEBA-175 F2 is in purple/light purple, the regions of PfEBA-175 outside RII are in dark blue, R217 light and heavy chain are in pink and slate blue respectively, R218 light and heavy chain are in orange and blue respectively, the IgG Fc is shown in white and lipid membranes are colored in light blue and yellow. (TIFF) [file ppat.1003390.s005.tif]
