# Supplementary material for: Structural and Functional Basis for Inhibition of Erythrocyte Invasion by Antibodies that Target Plasmodium falciparum EBA-175
Source: PLoS Pathog. 2013 May 23;9(5):e1003390. doi: 10.1371/journal.ppat.1003390 (PMC3662668; doi:10.1371/journal.ppat.1003390)
Supplement: Table S1 — Data collection and refinement statistics (PDF) [file ppat.1003390.s006.pdf]

Table S1

	RII-175/R217	F1/R218
**Data collection**		
Space group	C2	P2
Cell dimensions		
a, *b*, *c* (Å)	208.10, 101.26, 117.59	101.96, 53.53, 156.79
α, β, γ (°)	90.00, 102.86, 90.00	90.00, 91.184, 90.00
Resolution (Å) *	20.0-4.5 (4.6-4.5)	20.0-2.5 (2.6-2.4)
R_{sym} *	0.160 (0.412)	0.109 (0.942)
$I/\sigma I$ *	7.16 (1.92)	13.42 (1.90)
Completeness (%) *	92.8 (52.8)	98.8 (95.1)
Redundancy *	3.6 (2.1)	4.7 (4.7)
**Refinement**		
Resolution (Å)	20.0-4.5	20.0-2.45
No. reflections	13,315	62,597
$R_{work/}$ R_{free}	23.10/28.47	20.41/25.30
No. atoms		
Protein	17,565	11,563
Ligand/ion	0	60
Water	0	171
B-factors		
Protein	145.61	82.61
Ligand/ion	0	113.71
Water	0	62.51
R.m.s deviations		
Bond lengths (Å)	0.003	0.002
Bond angles (º)	0.662	0.609

Data were collected from a single crystal for each structure.
*Highest resolution shell is shown in parenthesis.
