# Supplementary material for: Structural and Functional Basis for Inhibition of Erythrocyte Invasion by Antibodies that Target Plasmodium falciparum EBA-175
Source: PLoS Pathog. 2013 May 23;9(5):e1003390. doi: 10.1371/journal.ppat.1003390 (PMC3662668; doi:10.1371/journal.ppat.1003390)
Supplement: Table S2 — RII-175/R217 interface residues defined by PISA [51] (PDF) [file ppat.1003390.s007.pdf]

Table S2

Residues in RII	Interface with R217 chains
K333	Light
P335	Heavy
Y336	Heavy
K337	Light, Heavy
L338	Light, Heavy
S339	Heavy
T340	Heavy
K341	Heavy
N417	Heavy
D418	Heavy
L419	Light, Heavy
R422	Light, Heavy
K423	Light
K439	Heavy
E545	Heavy

Residues in R217 Light Chain
S30A
Y30B
F32
H34
Y49
L50
H53
N91
N92
F96

Residues in R217 Heavy Chain
G26
Y27
T28
T30
E31
N32
T33
H35
N52
T52A
D53
N54
K73
T94
G95
Y96
D97
A98
M99
