# Supplementary material for: Structural and Functional Basis for Inhibition of Erythrocyte Invasion by Antibodies that Target Plasmodium falciparum EBA-175
Source: PLoS Pathog. 2013 May 23;9(5):e1003390. doi: 10.1371/journal.ppat.1003390 (PMC3662668; doi:10.1371/journal.ppat.1003390)
Supplement: Table S3 — F1/R218 interface residues as defined by PISA [51] (PDF) [file ppat.1003390.s008.pdf]

Table S3

Residues in F1	Interface with R218 chains
E149	Light, Heavy
W152	Heavy
E153	Heavy
L156	Heavy
S157	Heavy
K160	Heavy
N161	Heavy
N162	Heavy
I163	Heavy
N164	Light, Heavy
N165	Light
K167	Light, Heavy
N168	Light, Heavy
I169	Light, Heavy

Residues in R218 Light Chain
S30
Y32
Y50
R53
G91
S92
T93
F94
W96

Residues in R218 Heavy Chain
D31
N32
Y33
R50
D52
N54
N56
K58
Y96
G98
Y99
F100
L100A
Y100B
